# Chlorine disinfectant significantly changed microfauna habitat, community structure, and colonization mode in wastewater treatment plants

**DOI:** 10.1128/aem.01517-24

**Published:** 2024-12-20

**Authors:** Yuening Zhong, Yibo Zhang, Qiyue Meng, Haoyu Zhang, Zhenbing Wu, Chenyuan Dang, Jie Fu

**Affiliations:** 1 Hubei Key Laboratory of Multi-media Pollution Cooperative Control in Yangtze Basin, School of Environmental Science and Engineering, Huazhong University of Science and Technology 593423 , Wuhan, China; 2 Green Energy Industry Research Centre (GEIRC), Huazhong University of Science and Technology 12443 , Wuhan, China; Colorado School of Mines, Golden, Colorado, USA

**Keywords:** COVID-19 pandemic, chlorine disinfectant, activated sludge, microfauna community, protozoa

## Abstract

During the coronavirus disease 2019 epidemic, excessive chlorine disinfectants have been used to block the spread of severe acute respiratory syndrome-coronavirus 2, resulting in large amounts of residual disinfectants entering wastewater treatment plants (WWTPs) through sewage systems. So far, no relevant research has been conducted on the impact of chlorine disinfectants on microfauna, an important microbial component in activated sludge treatment systems. This study comprehensively investigated the changes in microfauna habitat, community structure, and colonization mode under the chlorine stress by combining the full-scale WWTP survey and laboratory-scale sequencing batch reactor experiments. The results showed that chlorine disinfectants significantly changed the community structure of microfauna, including decrease in sedentary ciliates and increase in free-living ciliates, amoebas, and flagellates. Besides the disinfection effect of chlorine disinfectants, the microfauna community was also influenced by changes in habitat and bacterial community. The loose structure and excessive extracellular polymeric substance (EPS) of activated sludge caused by chlorination would impact the colonization of sedentary ciliates, while it was conducive to the survival of free-living ciliates due to their predation on EPS as the nutrients. Bacteria in the activated sludge had strong interactions with protozoa, and their changes under chlorine stress directly affected the protozoan community and even indirectly affected the micro-metazoa community through the food chain.

**IMPORTANCE:**

This study revealed that chlorine disinfectant significantly changed microfauna habitat, community structure, and colonization mode in wastewater treatment plants during the coronavirus disease 2019 pandemic. Chlorine disinfectant could destroy the structure and stability of sludge flocs, reduce the abundance of beneficial microfauna in activated sludge, and even affect the colonization of sedentary ciliates on sludge. In addition, chlorine disinfectants might induce environmental and ecological risks related to microfauna, such as elevated suspended solids and release of bacteria and microfauna in the effluents.

## INTRODUCTION

Activated sludge treatment system is the most widely utilized biological treatment method (1). Activated sludge consists of a variety of microorganisms such as bacteria, archaea, eukaryotes (fungi, algae, protozoa, and micro-metazoa), and viruses (such as bacteriophages). Bacteria are the main component of the microbial community in activated sludge, but as the main predators of bacteria, protozoa and micro-metazoa also play important roles in the biological process of activated sludge (2). Protozoa can reduce the concentration of suspended particles in effluent by predation of free-living bacteria (3), and the substances released by the protozoa can promote the flocculation and sedimentation of sludge flocs (4). In addition, the growth and reproduction of bacteria can also be stimulated by protozoa predation (5), which plays an important role in facilitating the remineralization of nutrients and carbon respiration (6–8). Micro-metazoa often promote sludge settlement through predation (8), improve sludge dewatering performance, and expedite the diffusion of oxygen and nutrients in activated sludge through their mechanical actions (9). The community succession of microfauna indicates the stability and efficiency of activated sludge treatment system in some ways. For example, *Vorticella* and *Epistylis* often appear in the system with good effluent quality, while flagellates and amoebas often appear frequently in the system with poor effluent quality (10–13), and nematodes generally indicate high sludge load (14). At the same time, the stable food chain relationship between protozoa and micro-metazoa is similar to the “synergistic” relationship to some extent (15), which is of great significance for improving the effluent quality of activated sludge system.

Microfauna in activated sludge are highly sensitive to toxic substances, and many traditional pollutants (e.g., heavy metals, chlorine disinfectants) and emerging pollutants (e.g., microplastics, persistent organic pollutants) have strong toxic effects on microfauna (16), which can significantly alter their community structure. Since the beginning of 2020, the coronavirus disease 2019 (COVID-19) has swept the world, causing huge damage to society, economy, environment, and human health, which is considered to be one of the most serious infectious diseases in the past century (17). During the COVID-19 pandemic, excessive chlorine disinfectants have been used in medical institutions and public spaces to block the spread of the severe acute respiratory syndrome-coronavirus 2, resulting in a large number of chlorine disinfectants poured into sewage systems and even drinking water resources (18, 19). For example, the residual chlorine was detected in the concentration range of 0–0.22 mg/L in the influents of eight wastewater treatment plants (WWTPs) in Wuhan in February 2020. The previous study has found that chlorine disinfectants could significantly alter the bacterial community structure and function in activated sludge, thereby reducing the removal of carbon and nitrogen from wastewater by bioreactors (18). However, no relevant research has been conducted on the impact of chlorine disinfectants on microfauna in activated sludge and the corresponding contribution to the functional decline of bioreactors.

Some studies have shown the toxic effects of chlorine disinfectants on protozoa, but the response varies among different species. For example, Daw et al. found that high concentrations of sodium hypochlorite can lead to the death of ciliate *Euplotes* (20), but there were also cases where amoebas or some flagellates could resist the toxicity of low-dose chlorine (21). Considering that the impact of the COVID-19 on the environment is lasting and permanent, it is necessary to find out whether the chlorine disinfectants used extensively during the epidemic will affect the microfauna in activated sludge and whether different groups of microfauna have specific responses to chlorine toxicity.

Therefore, we surveyed the diversity of microfauna in activated sludge of a full-scale WWTP in Wuhan before and after the COVID-19 (2019–2021), as well as the residual chlorine in influents and the treatment performance of bioreactors. Based on the investigation results, the laboratory-scale reactor experiments were further conducted, and the changes of effluent quality, sludge characteristic, bacterial and microfauna communities, and colonization mode of microfauna under chlorine stress were comprehensively studied. The research objectives were to reveal (i) the changes of sludge habitat under chlorination; (ii) the effects of chlorination on the growth, colonization, and succession of microfauna; (iii) the relationship between habitat and microfauna; and (iv) the interaction between bacteria and microfauna.

## MATERIALS AND METHODS

### Survey on WWTP

The surveyed WWTP (Wuhan, China) has a treatment scale of 0.1 million tons per day. The used biochemical process is oxidation ditch process. The water quality data (water inflow, residual chlorine in influent, chemical oxygen demand (COD), ammonium nitrogen, total phosphorus) and microscopic photographs in February of 2019–2021 were collected and analyzed. Water quality data were tested once a day.

### Reactor start-up and operation

The active sludge was taken from a WWTP (Wuhan, China). After sludge domestication by feeding with synthetic wastewater, four sequencing batch reactors (SBRs, denoted as control, eg1, eg2, and eg3) had been conducted at room temperature. The SBR reactors had a working volume of 1 L. A mechanical stirrer was applied for liquid mixing in the SBRs to maintain the dissolved oxygen greater than 2 mg/L. The specific composition of synthetic wastewater is provided in Table S1. The operation cycle of SBR was 24 h, consisting of 5 min feeding, 23 h continuous stirring and aeration, 50 min sludge precipitation, and 5 min drainage, and the exchange volume of each reactor was 500 mL per day. The initial mixed liquor suspended solids (MLSS) in each reactor was ~3500 mg/L. Four doses of NaClO (0 mg/L, 1 mg/L, 2 mg/L, and 6 mg/L) were added into inlet waters to simulate the chlorinated wastewater, and after reacting for half an hour, four doses of residual chlorine (free chlorine) were obtained in the inlet waters of four reactors (control: 0 mg/L, eg1: 0.08 mg/L, eg2: 0.11 mg/L, and eg3: 0.32 mg/L). The water quality parameters of chlorinated wastewater are shown in Table S2. The experimental period lasted for 30 days, and chlorination experiment was conducted on days 1–20, and restoration experiment without addition of NaClO was conducted on days 21–30.

### Water quality parameter analysis

Water quality parameters of reactor influents and effluents were measured every day. Chemical oxygen demand, ammonium nitrogen (NH_4_^+^-N), nitrite nitrogen (NO_2_^−^-N), nitrate nitrogen (NO_3_^−^-N), free chlorine, and total suspended solids (TSS) were measured by the standard methods (22). The bacterial number in the effluents was estimated by heterotrophic plate count (HPC) method, which was described previously (23).

### Physicochemical analysis of activated sludge

Activated sludge samples were collected at the predetermined time. The morphology of sludge was observed by scanning electron microscopy (SEM, JSM-IT200, JEOL, Japan), and the sludge treatment procedures are described in Text S1. The particle size distribution of sludge was determined by a laser particle size analyzer (Masterizer-2000, Malvern, UK). Sludge sedimentation performance was represented by sludge volume index (SVI) measured at 30 min. The zeta potential of the sludge was determined by a potential analyzer (Nano-ZS90, Malvern, UK).

### EPS extraction and analysis in activated sludge

Extracellular polymeric substance (EPS), including loosely bound EPS (LB-EPS) and tightly bound EPS (TB-EPS), in activated sludge was extracted according to the reported method (24), and detailed procedures are described in Text S2. The contents of polysaccharide (PS) and protein (PN) in EPS were determined by modified Lowry method and phenol-sulfuric acid method, respectively. Fluorescence excitation-emission matrix (EEM) spectra of the sludge EPS were measured by using a fluorescence spectrometer (F-7100, Hitachi, Japan).

### DNA extraction and sequencing

Sludge samples were collected on days 0, 1, 20, and 30 from the four SBR reactors. The samples collected on day 0 were mixed as the initial sludge. FastDNA Spin Kit for Soil (MP Biomedicals, USA) was used to extract the total microbial DNA of each sludge sample according to manufacturer’s protocols. DNA samples were amplified using the GeneAmp PCR System 9700 (ABI, USA) for full-length (V1–V9 regions) 16S/18S rRNA genes. After purification by electrophoresis, the amplicons were sequenced on the PacBio Sequel platform (Biozeron Company, Shanghai, China). The detailed procedures for PCR amplification and sequencing are described in Text S3.

### Sequencing data analysis

SMRT Link Analysis software (v. 9.0) was used to process the PacBio raw reads. Demultiplexed circular consensus sequence was obtained, and sequences in a range of <700 bp or >1500 bp were removed. UCLUST algorithm was used to cluster operational taxonomic units (OTUs) with a 98.65% similarity cutoff. Each OTU was classified into domain, phylum, class, order, family, genus, and species according to SILVA database. Taxonomic diversity analysis was performed to assess delta diversity using PRIMER software (v. 7.0, Prime-E, UK). Hierarchical clustering analysis of taxa was based on Bray–Curtis dissimilarity using unweighted pair-group method with arithmetic means. FAPROTAX was used to analyze and predict the function of bacterial community. Procrustes analysis was performed by using the R package vegan, and the consistency of the evolution of taxonomic diversity of bacteria and protozoa was analyzed, visualizing by the coordinate diagram of principal coordinate analysis. The correlation of evolution between bacteria, protozoa, and micro-metazoa was explored using network analysis and visualized in Gephi software (v. 0.10.1).

### Colonization experiment of isolated *Vorticella* on activated sludge

The isolation and purification of *Vorticella* from activated sludge were according to the reported methods (25, 26) with modifications. In brief, 2 mL of activated sludge was extracted from an SBR reactor into the Petri dish. A 30 mL wheat extract (1 g/L) was added with exchange every 2 days, and the Petri dish was placed at 20 ± 1℃ for 4–10 days, ensuring a small number of *Vorticella* individuals appeared at the bottom of the Petri dish. Then the sludge was removed from the Petri dish, and the bottom of the dish was rinsed reverse osmosis water for 2–3 times. After a series of purification steps, the pure *Vorticella* were obtained, and their cells were cultured in an inorganic medium (0.24 mM MgSO_4_, 0.24 mM NaCl) with wheat extract (9:1). Specific purification methods are described in Text S4.

For the colonization experiment, two types of activated sludge were used, i.e., unchlorinated sludge (treated with no chlorine disinfectant) and chlorinated sludge (treated with 2 mg/L chlorine disinfectant). A 2 mL mixture of activated sludge and 25 *Vorticella* cells was poured into a Petri dish. The experiment was conducted for 3 days, and the colonization of *Vorticella* on the sludge was observed through a microscope.

## RESULTS

### Change of microfauna in activated sludge of a full-scale WWTP in Wuhan before and after COVID-19 (2019–2021)

The water quality parameters of influents and effluents of the WWTP in February of 2019–2021 are summarized in Table S3. During the COVID-19 epidemic period (February 2020), the residual chlorine was detected in the influents with an average concentration of 0.12 mg/L, while before the epidemic (February 2019) or in mitigated period (February 2021), no residual chlorine was detected. Therefore, this result can prove that high-dose disinfection during the COVID-19 directly caused the residual chlorine disinfectants to enter WWTPs (19). The removal of main pollutants had shown a slight reduction in 2020, especially the removal of total phosphorus, which decreased by 4.98% and 6.02% compared to 2019 and 2021, respectively.

**Fig 1:**
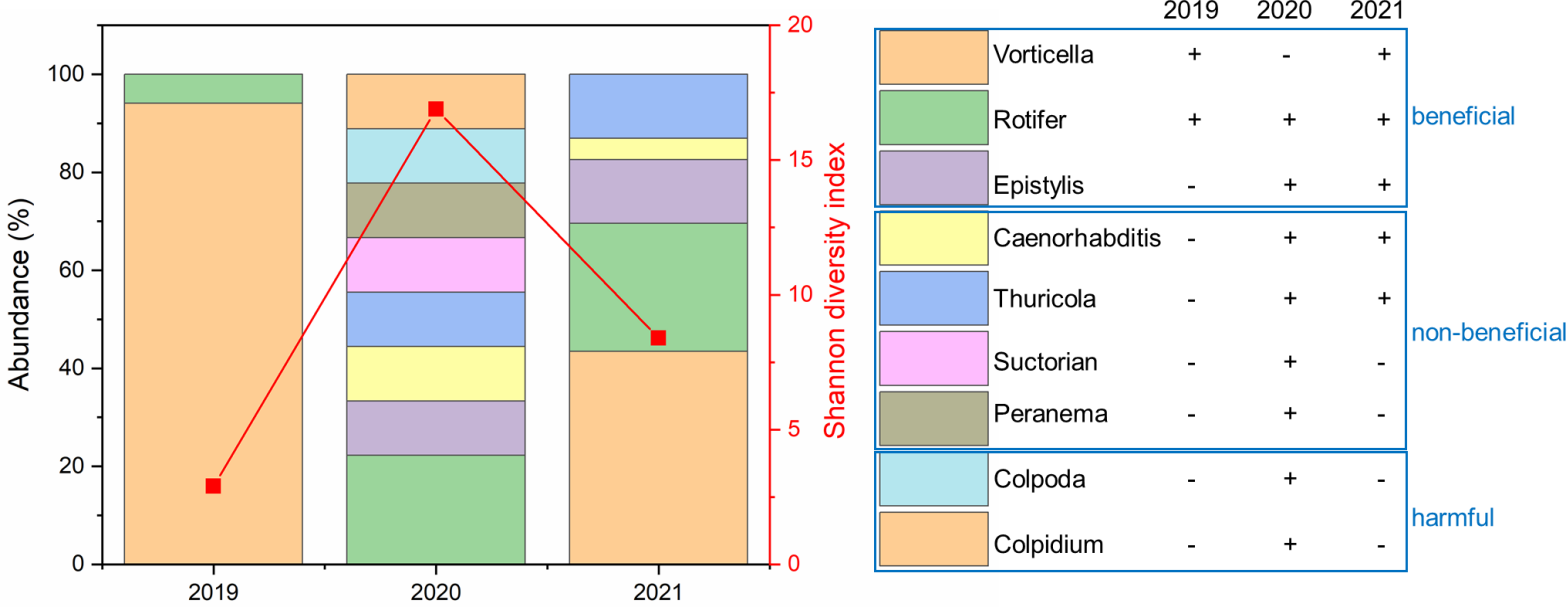
Change of microfauna in the activated sludge of a full-scale WWTP in Wuhan in February of 2019–2021.

In terms of microfauna in the activated sludge, before the COVID-19 in 2019, the species diversity of protozoa/micro-metazoa was very low (Shannon diversity index = 2.89), and only beneficial microfauna such as *Vorticella* and rotifers had been found (Fig. 1), suggesting a stable biological treatment system (13). However, with the outbreak of COVID-19 in 2020, the Shannon diversity index increased significantly to 16.88, and large numbers of non-beneficial and harmful microfauna occurred. Clearly, the large amount of residual chlorine entering significantly destroyed the stability of the sludge microecosystem. In 2021, with the easing of the epidemic and the decrease in the use of chlorine disinfectants, the species diversity of microfauna gradually decreased with a Shannon diversity index of 8.40.

### Effects of chlorine disinfectant on microfauna habitat in SBR reactors

**Fig 2:**
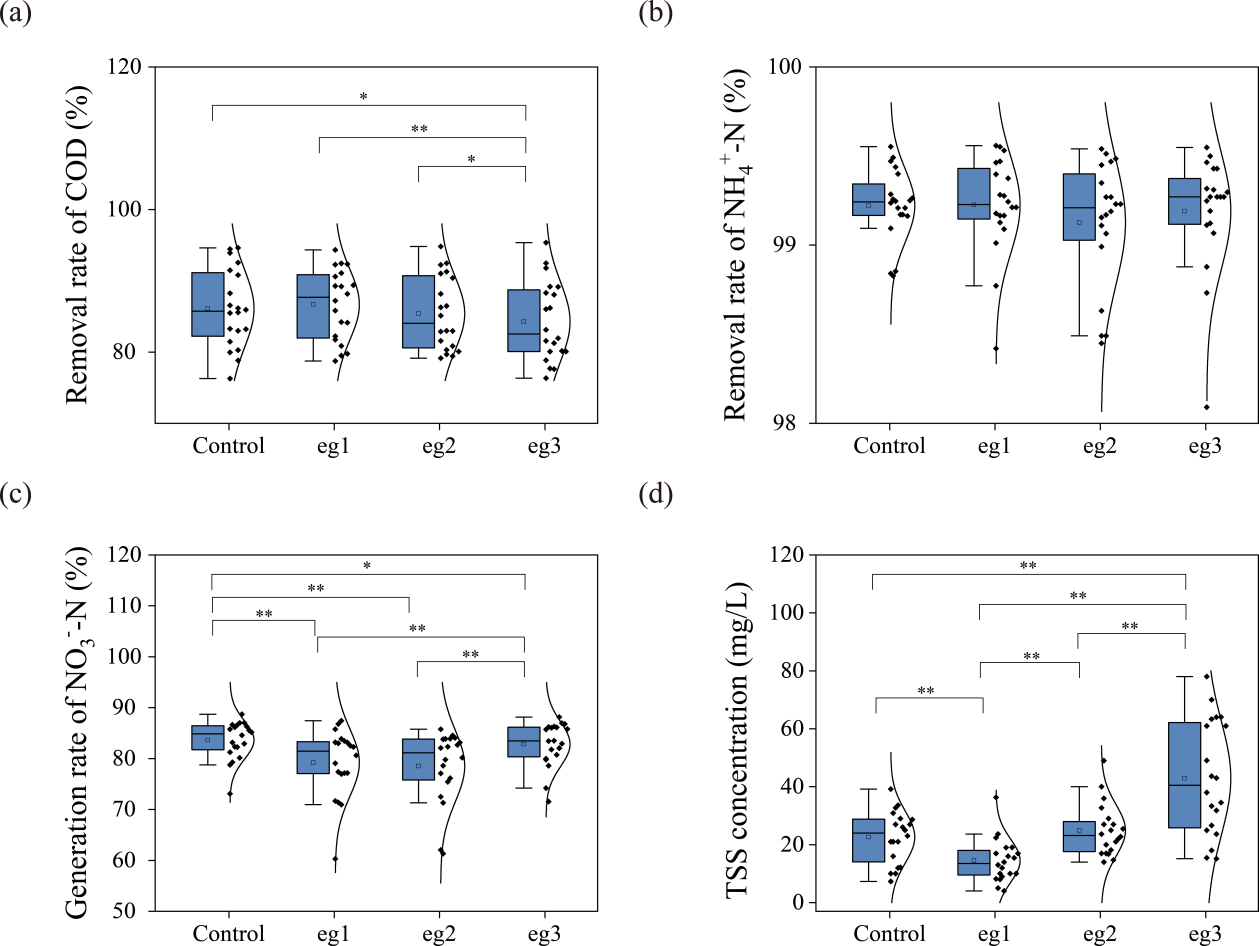
Effects of chlorine disinfectant on treatment performance of SBR reactors. Removal rate of COD (**a**) and NH_4_^+^-N (**b**), generation rate of NO_3_^−^-N (**c**), and TSS concentration in effluents (**d**). Significantly different when compared two groups was marked with * at *P* < 0.05 level and ** at *P* < 0.01 level.

Figure 2 summarizes water quality of effluents during chlorination period in SBR experiments. The addition of high-dose chlorine disinfectants in eg3 significantly reduced the removal rate of COD (Fig. 2a), suggesting that high-dose chlorine disinfection might impact microbial carbon metabolism (18). For the removal of NH_4_^+^-N (Fig. 2b), chlorine disinfectants seemed to have little impact on it. However, the generation of NO_3_^−^-N was significantly inhibited (Fig. 2c). As is well known, chlorine disinfectants can inhibit the activity of nitrifying bacteria (27), which will reduce the removal of NH_4_^+^-N and generation of NO_3_^−^-N. In addition, chlorine disinfectants have strong oxidizing capacity and can react with ammonia to produce chloramines (28) and even further oxidize to nitrite or nitrate at high dose (29), which would compensate partial reduction in nitrification. It is worth noting that the addition of high-dose chlorine disinfectants resulted in the significant elevation of TSS in the SBR effluents (Fig. 2d). This suggests that the residual chlorine would destroy the flocculent structure of activated sludge by its strong oxidative capacity (30). Consequently, the concentration of activated sludge (MLSS) in the reactors was also reduced by the chlorination (Table S4).

**Fig 3:**
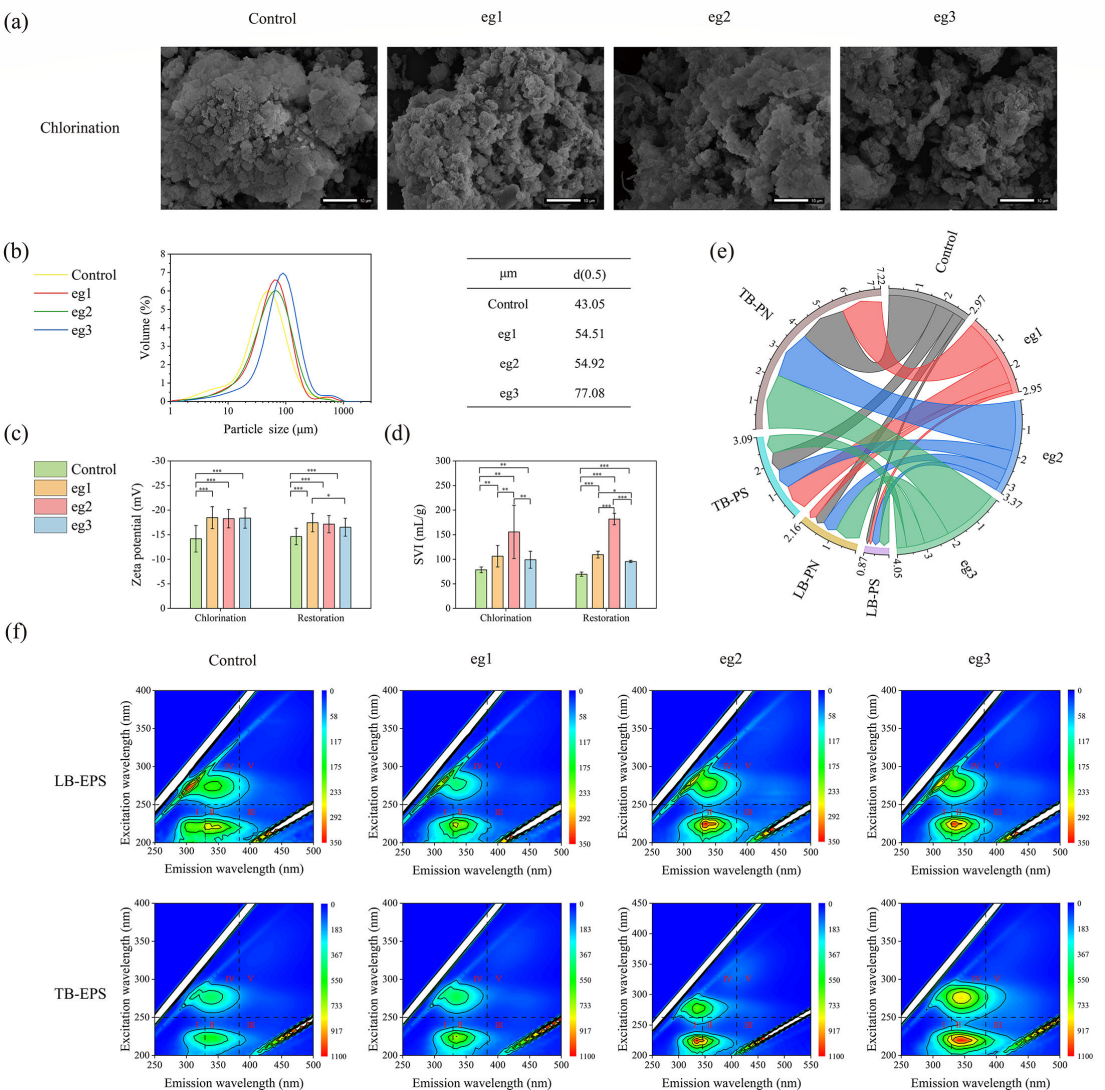
Effects of chlorine disinfectant on physicochemical properties of activated sludge in SBR reactors. (**a**) SEM images of sludge flocs during chlorination period. The length of each scalebar is 10 µm. (**b**) Particle size distribution and median size of sludge flocs. (**c**) Zeta potential of sludge flocs. (**d**) SVI index of sludge flocs. (**e**) PS and PN content of sludge EPS in each reactor (mg/g·MLSS). Significantly difference when compared two groups was marked with * at *P* < 0.05 level, ** at *P* < 0.01 level, and *** at *P* < 0.001 level. (**f**) EEMs of LB-EPS and TB-EPS during chlorination period (ex: 200–450 nm, em: 250–500 nm). Region I, aromatic protein I (tyrosine); region II, aromatic protein II (tryptophan); region III, fulvic acid; region IV, soluble microbial by-product; and region V, humic acid.

To study the effect of chlorine disinfectant on microfauna habitat, the physicochemical analyses of activated sludge were conducted. According to the SEM image (Fig. 3a), with the increase of chlorine concentration, the flocs of the sludge showed a loose structure, and the gaps of the sludge increased. The median size of sludge flocs in four reactors was 43.05 µm, 54.51 µm, 54.92, and 77.08 µm, respectively, showing an increasing trend (Fig. 3b). Zeta potential is an index to measure the charging characteristics of sludge surface (31). With the addition of chlorine disinfectants in the influents, the absolute value of zeta potential of activated sludge significantly increased (Fig. 3c), indicating a potential decline of flocculation/sedimentation of sludge flocs. As expected, the sedimentation of activated sludge deteriorated as the chlorine dose increased from 0 to 2 mg/L (Fig. 3d). However, as the chlorine dose continued to increase to 6 mg/L, the sedimentation of activated sludge accelerated again. A possible explanation is that adding a high concentration of electrolyte (NaClO) could promote the sludge sedimentation by compressing the thickness of the electric double layer surrounding the sludge flocs (32).

For the sludge EPS, the chlorination promoted its secretion. As shown in Fig. 3e, with the increase of chlorine concentration, the EPS concentration (PS+PN content) in each reactor increased, which were 2.97, 2.95, 3.37, and 4.05 mg/g·MLSS, respectively. The EEM also exhibited an evident increase in fluorescence intensity with the increase of chlorine concentration (Fig. 3f), especially in regions I (tyrosine) and II (tryptophan) (33). These results indicate that secretion of EPS was the typical measure for microorganisms (mainly bacteria) in activated sludge to alleviate the chlorine stress (18).

### Effects of chlorine disinfectant on bacterial community in activated sludge

**Fig 4:**
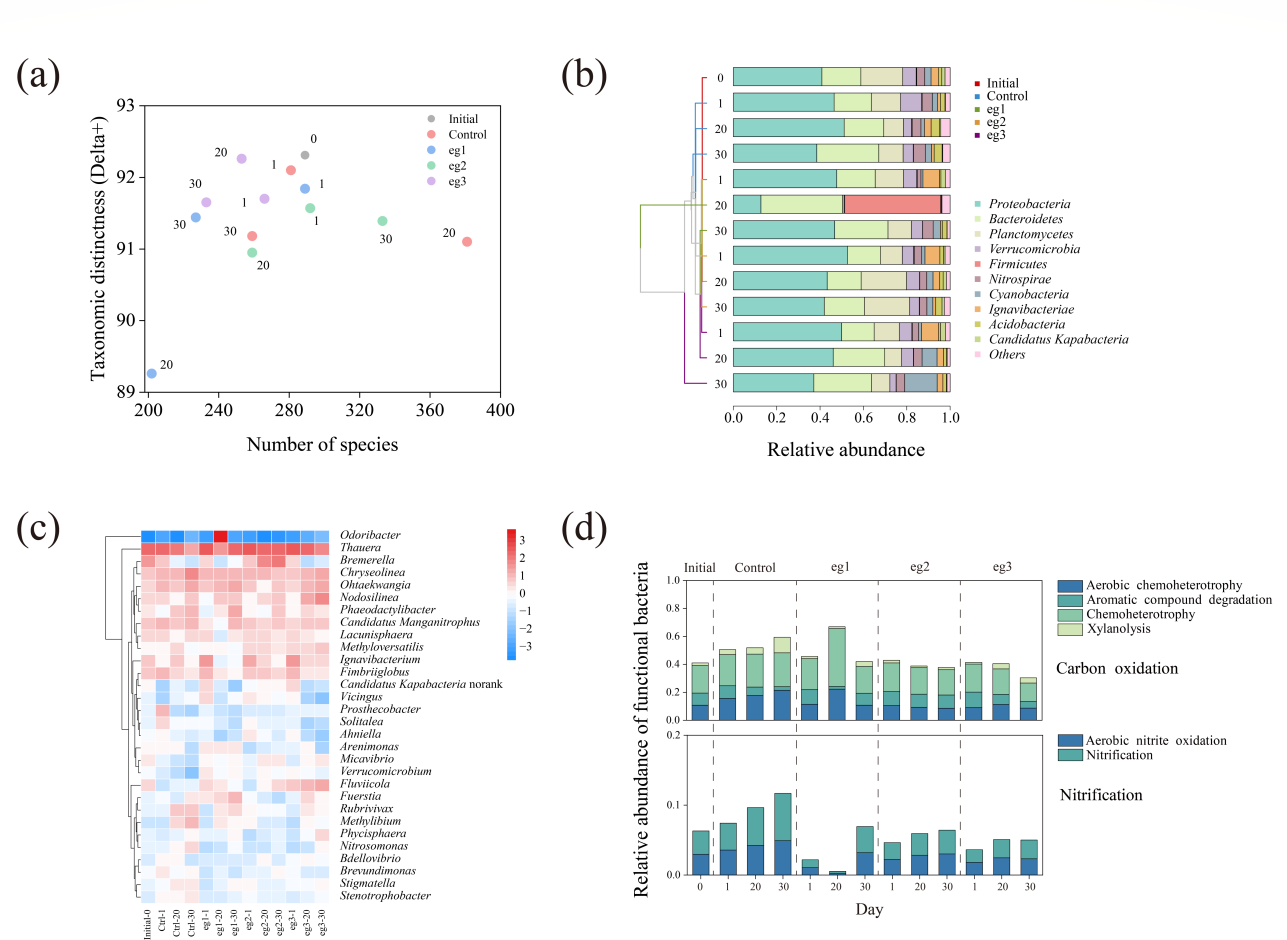
Effects of chlorine disinfectant on bacterial community in activated sludge. (**a**) Bacterial taxonomic diversity in each sludge sample. (**b**) Relative abundance of bacteria at the phylum level with hierarchically clustering based on Bray–Curtis dissimilarity. (**c**) Heatmap of the relative abundance of bacteria at the genus level (top 30). The raw data were logarithmically processed with a base of 2. (**d**) Relative abundance of functional bacteria related to carbon oxidation and nitrification. The top 100 bacterial species (accounted for 81.5% of the total relative abundance) were assigned to the specific functional bacteria by FAPROTAX.

Figure 4a shows the bacterial taxonomic diversity in each sludge sample. Typically, except the sample eg1–20, the taxonomic distinctness values of all the samples were close and distributed in a very narrow range (90.5–92.5), indicating a relatively stable taxonomic diversity (biodiversity) of bacterial community. However, the addition of chlorine disinfectants significantly reduced the species number (species diversity).

To investigate the specific bacterial taxa affected by the chlorine stress, the bacterial community structure was analyzed at phylum and genus levels. As shown in Fig. 4b, a total of 33 bacterial phyla were found, and *Proteobacteria* (12.7%–52.6%) and *Bacteroidetes* (15.0%–37.7%) were the dominant ones. The relative abundance of *Proteobacteria* visibly increased in the initial stage of chlorination (day 1) but gradually decreased with the prolonging of exposure time. At the genus level, a total of 670 bacterial genera were found, and the heatmap of relative abundance was drawn for the first 30 genera (Fig. 4c). The dominant genera included *Thauera* (0.87%–35.74%), *Chryseolinea* (0.38%–11.63%), and *Bremerella* (0.01%–14.29%), *Ohtaekwangia* (0.42%–6.87%), and *Nodosilinea* (0.31%–14.97%). Overall, these dominant bacteria exhibited resistance to chlorine disinfectants, manifested by an increase in relative abundance under chlorine stress.

Furthermore, the effects of chlorine disinfectant on the functional bacteria related to carbon oxidation and nitrification were investigated (Fig. 4d). It can be seen that with the increase of chlorine concentration, carbon oxidation capacity and nitrification capacity were evidently inhibited, which was the same trend as the effluent quality (Fig. 2a and c).

### Effects of chlorine disinfectant on microfauna community in activated sludge

**Fig 5:**
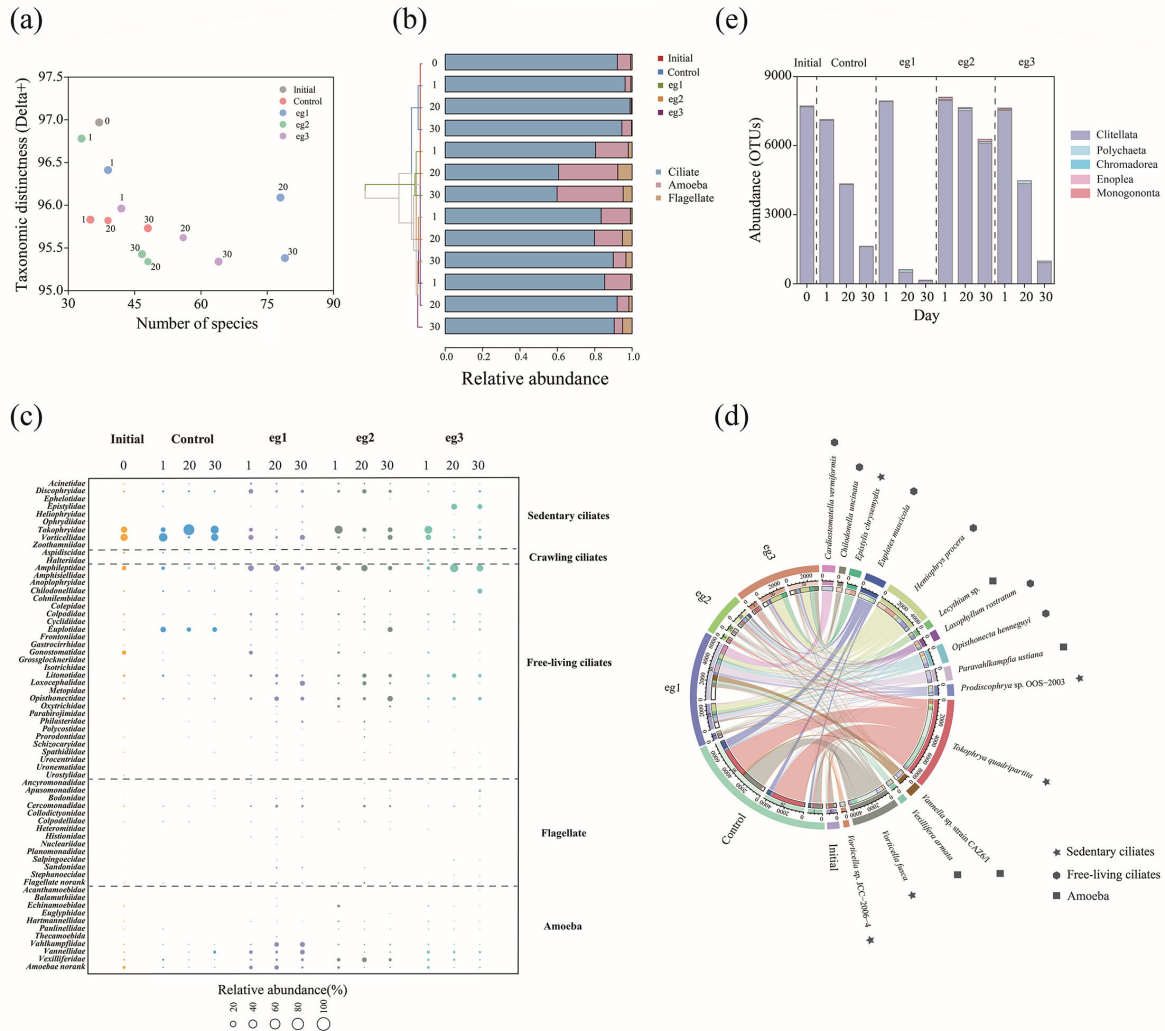
Effects of chlorine disinfectant on microfauna community in activated sludge. (**a**) Protozoa taxonomic diversity in each sludge sample. (**b**) Relative abundance of protozoa at the categories of ciliates, amoebas, and flagellates with hierarchically clustering based on Bray–Curtis dissimilarity. (**c**) Bubble chart of the relative abundance of protozoa at the family level. (**d**) Chord diagram showing the distribution of top 15 protozoa species (on the right) in the sludge samples (on the left). The length of each arc represents the number of specific OTUs. The sedentary ciliates, free-living ciliates, and amoebas are labelled with star, polygon, and square, respectively. (**e**) Accumulative histogram of abundance of micro-metazoa at class level.

Compared with the bacterial community, the protozoa in the activated sludge showed a relatively higher taxonomic diversity with an average taxonomic distinctness value of 95.90 ± 0.45 (Fig. 5a). The protozoa community in the control group was relatively stable, while the addition of chlorine disinfectants significantly increased the species number, resulting in the fluctuation of taxonomic diversity in experimental groups. After removing the chlorine stress during the restoration period, the increasing trend of the species number slowed down, indicating a strong driving effect of chlorine disinfectants on protozoa community.

To explore the effect of chlorine disinfectants on the growth and living habits of protozoa in activated sludge, protozoa were divided into three categories, i.e., ciliates, amoebas, and flagellates (Fig. 5b). In the control group, ciliates were the dominant protozoa, accounting for 96.57 ± 2.21% in relative abundance. With the addition of chlorine disinfectants, the relative abundance of ciliates significantly decreased, and instead those of amoebas and flagellates increased. Even after removing chlorine disinfectants in the restoration period, the abundance of ciliates still had not recovered. These results demonstrated the destructive effect of chlorine disinfectants on protozoa community, particularly the inhibitory effect on ciliates. Interestingly, the relative abundance of amoebas and flagellates increased more in eg1 (low-dose group, by 38.20% in sample eg1–20 compared with sample control-20) than in eg2 (medium-dose group, by 19.02%) and eg3 (high-dose group, by 6.89%). This indicated that high-dose chlorine disinfectants also had disinfection effect on amoebas and flagellates, and some ciliates might show the chlorine resistance in some extent.

In terms of taxonomic information, the response of protozoa community to chlorine disinfectants was analyzed at the family level (Fig. 5c). The ciliates were further divided into sedentary, crawling, and free-living ones according to their locomotion patterns. The sedentary ciliates (e.g., *Vorticellidae*) were the predominant protozoa in the control group (72.33 ± 11.65%), indicating a mature and stable status of activated sludge (34). The addition of chlorine disinfectant significantly reduced the abundance of sedentary ciliates in the experimental groups on day 20 (18.85 ± 9.93%), and the abundance of sedentary ciliates moderately restored in the restoration period on day 30 (28.34 ± 5.57%). In addition, it can be clearly seen that with the increase of chlorine concentration, the relative abundance of free-living ciliates (e.g., *Amphileptidae*) showed a remarkable growth, which can explain the trend of re-increase in ciliate abundance with high-dose chlorine disinfectants (Fig. 5b). For the amoebas and flagellates, the dominant families increased in the experimental groups included *Vexilliferidae* (4.45 ± 3.98%), *Vannellidae* (4.06 ± 4.30%), and *Vahlkampfiidae* (3.48 ± 5.89%).

The protozoa community was further analyzed at the genus and species levels, and a total of 99 genera and 143 species were found. The chord diagram (Fig. 5d) mapped the distribution of dominant protozoa species (top 15) in the sludge samples, including 5 sedentary ciliates (*Tokophrya quadripartite*, *Vorticella fusca*, *Prodiscophrya* sp. OOS-2003, *Epistylis chrysemydis*, and *Vorticella* sp. JCC-2006-4), 6 free-living ciliates (*Hemiophrys procera*, *Opisthonecta henneguyi*, *Euplotes muscicola*, *Loxophyllum rostratum*, *Cardiostomatella vermiformis*, and *Chilodonella uncinata*), and 4 amoebas (*Lecythium* sp., *Vexillifera armata*, *Vannella* sp. strain CAZ6/I, and *Paravahlkampfia ustiana*). The addition of chlorine disinfectants markedly decreased the total abundance of these dominant species, which contributed to the increase of diversity in protozoa community. As for specific species of sedentary ciliates, *Tokophrya quadripartite*, *Vorticella fusca,* and *Vorticella* sp. JCC-2006-4 decreased in abundance in experimental groups. In contrast, the abundance of *Epistylis chrysemydis* increased considerably in the high-dose group eg3. The previous study has also reported the chlorine resistance of *Epistylis* (35), and this might be related to their population growth pattern, which provided recalcitrance to the environmental stress. As for the specific species of free-living ciliates and amoebas, except *Euplotes muscicola*, the other dominant species all showed a growing trend with the exposure of chlorine disinfectants.

In addition to protozoa, activated sludge also contained some micro-metazoa. A total of five classes were found, including *Clitellata* (from phylum *Annelida*), *Polychaeta* (from phylum *Annelida*), *Chromadorea* (from phylum *Nematoda*), *Enoplea* (from phylum *Nematoda*), and *Monogononta* (from phylum *Rotifera*), in which the dominant micro-metazoa was *Clitellata* with an average relative abundance of 96.01 ± 5.52% (Fig. 5e). In the early stage of the chlorination (day 1), the chlorine disinfectant did not have much impact on clitellates, and the abundance of rotifers and nematodes increased in the medium- and high-dose groups, indicating a recalcitrance to chlorine disinfectants. With the progress of the experiment, the abundance of clitellates in all reactors decreased, but the degree of reduction in different groups varied. Compared with the control group, the abundance of clitellates showed a significant decrease in the low-dose group (eg1) but a remarkable increase in the medium- and high-dose groups (eg2 and eg3). This trend is similar to the change of ciliates (Fig. 5b), suggesting that predation between clitellates and ciliates may have played an important role for the distribution of clitellates in the activated sludge.

## DISCUSSION

### Interaction between habitat changes and microfauna community

**Fig 6:**
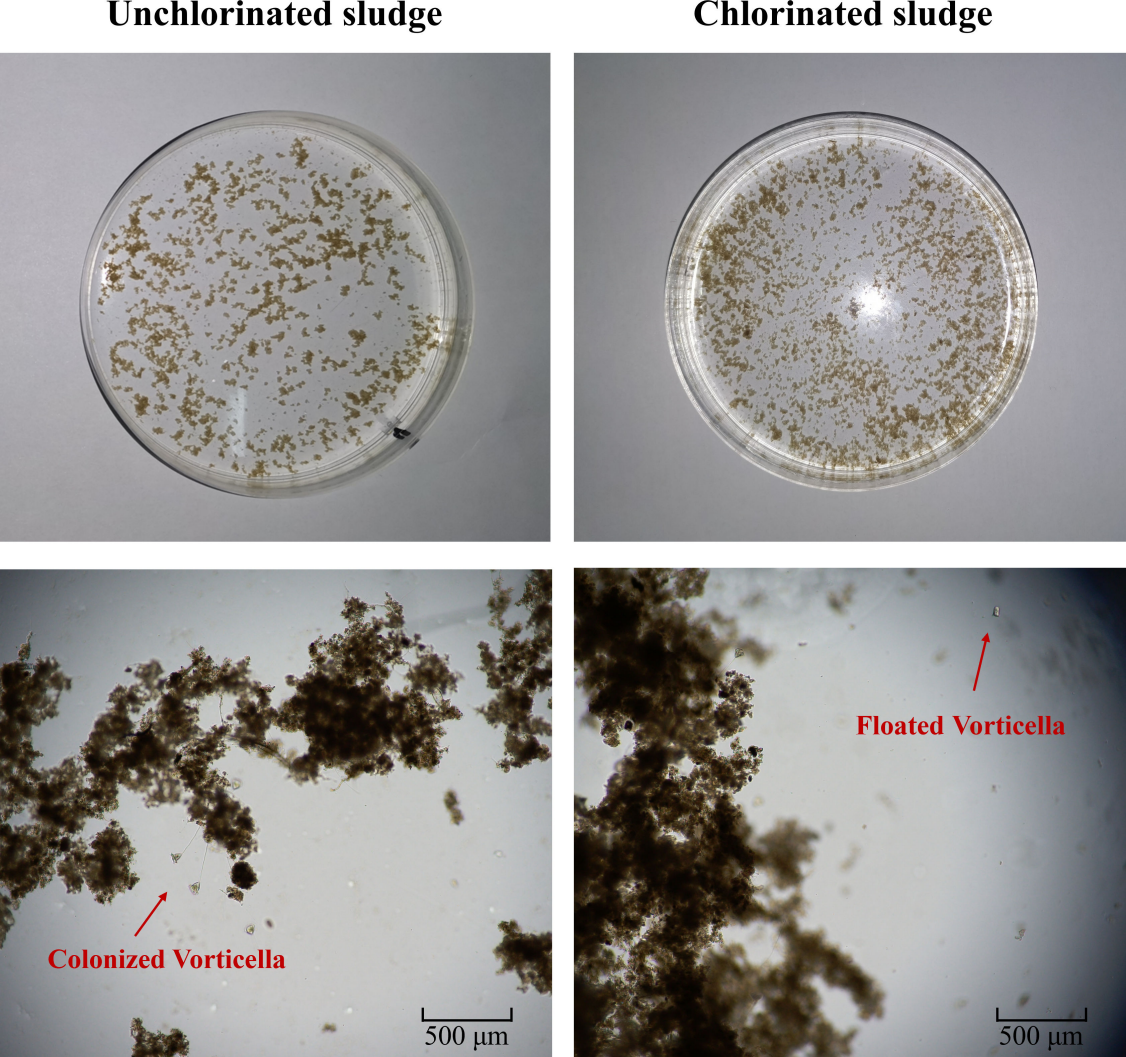
Colonization of isolated *Vorticella* on unchlorinated (left) and chlorinated (right) activated sludge.

Habitat characteristics can drive changes in community composition and diversity of microfauna community (36). The above results indicated that chlorination had greatly changed the characteristics of activated sludge, such as loosing the floc structure, increasing the particle size, elevating the zeta potential, deteriorating the sedimentation, and increasing the EPS content (Fig. 3). The loose structure and excessive EPS would decrease the compress strength of activated sludge (37), which seems to be unfavorable for the colonization of sedentary ciliates. In addition, the excessive secretion of EPS would cover the adsorption sites on the sludge surface (38), which is also not conducive to the colonization of sedentary ciliates. To demonstrate this hypothesis, we carried out the colonization experiment of isolated *Vorticella* on activated sludge treated by chlorination through microscope observation. As shown in Fig. 6, in the control group with the sludge being not treated by chlorination, the *Vorticella* could colonize and grow well on the activated sludge. However, in the treatment group with sludge being treated by chlorination, the *Vorticella* could not effectively colonize on activated sludge and could only survive in a floating state. Therefore, this result further confirms our previous speculation. For the free-living ciliates (e.g., *Hemiophrys*), they could prey on EPS as their own nutrition sources (39). Consequently, it is observed that the chlorination was more conducive to the surviving of free-living ciliates (Fig. 5c and d).

In turn, microfauna can also affect the characteristics of activated sludge to a certain extent. Previous studies (40) have shown that *Epistylis* can promote sludge flocculation and sedimentation, which may be one of the reasons why eg3 had a lower SVI than eg1 and eg2 (Fig. 3d). In addition, various studies (41–43) also found that the *Clitellata* could make the sludge floc loose by preying and ingesting the sludge, contributing to the increase of suspended particles in the effluent of eg3 (Fig. 2d). Moreover, the mass propagation of the *Clitellata* caused a certain disturbance to the sludge floc, leading to the increase of SVI (44).

### Interaction between bacteria and microfauna in activated sludge

**Fig 7:**
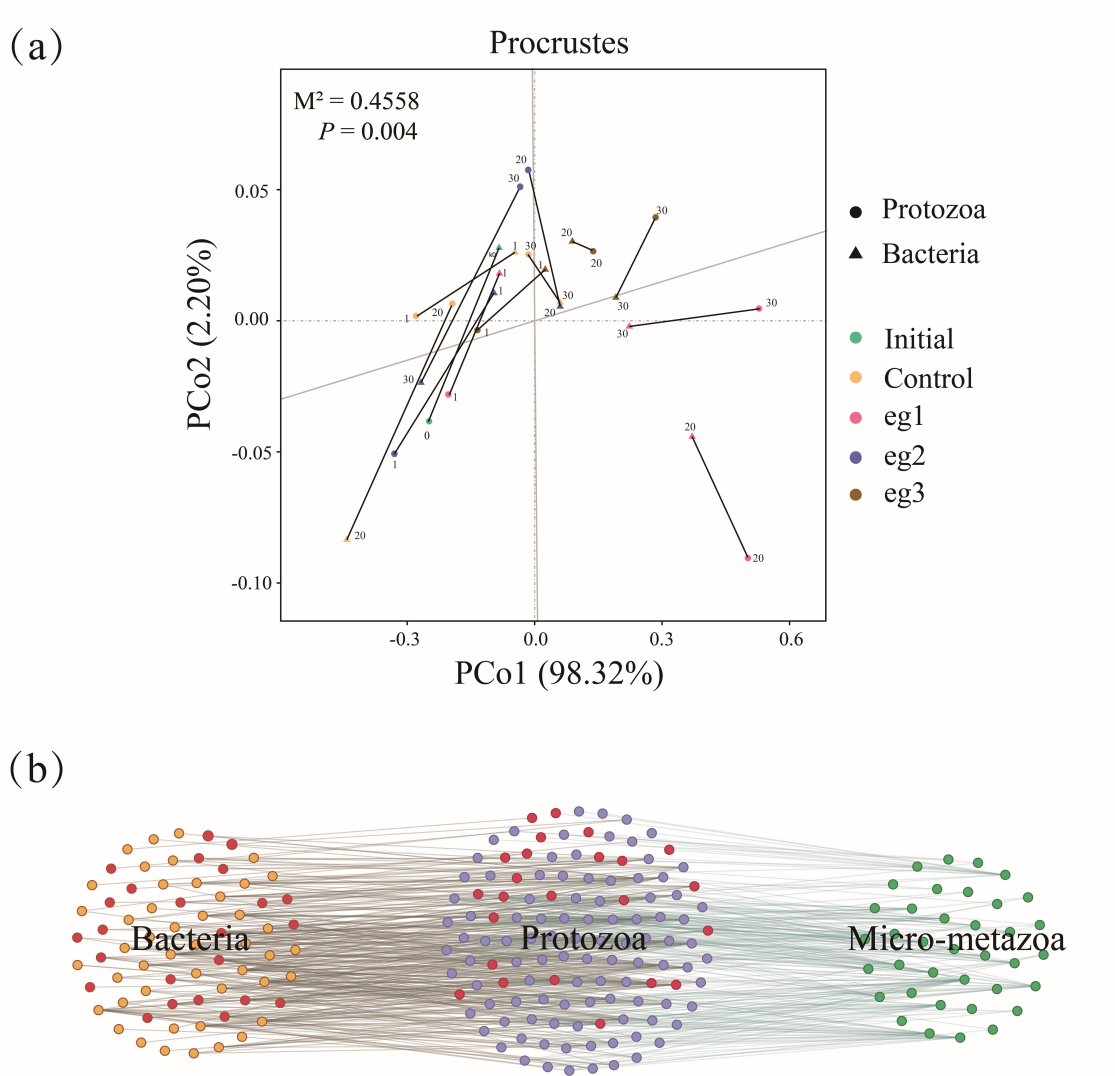
Interaction between bacteria and microfauna in activated sludge. (**a**) Procrustes analysis between bacterial community and protozoa community based on taxonomic distinctness and species number. (**b**) Network analysis on bacteria-protozoa-micro-metazoa correlation network. Significant interactions are at *P* < 0.01 level. The highlighted nodes in red color are functional bacteria related to carbon oxidation and nitrification and sedentary ciliates.

Bacteria-protozoa-micro-metazoa constitute the food chain in the microecosystem of activated sludge, so it is of great significance to study the correlation between the three communities. The overall correlation between bacterial community and protozoa community was analyzed by Procrustes analysis (Fig. 7a). The result showed that there was a significant consistency between the changes of bacterial community and protozoa community (M^2^ = 0.4558, *P* = 0.004). This suggest that besides the external chlorine stress, the biotic interactions, such as predation between bacteria and protozoa (45), can still be critical factors influencing the microbial community.

The network analysis on the top 100 bacteria species, all protozoa species (143), and micro-metazoa species (41) was carried out (Fig. 7b). There existed significant interactions between 70 bacteria and 81 protozoa (*P* < 0.01), including 68 positive interactions and 283 negative interactions. It is proved that there was a certain predation relationship between most bacteria and protozoa (39). All over the network, there are 24 functional bacteria involved in carbon oxidation and nitrification and 13 sedentary ciliates, but only 18 interactions were found among them. This might be related to the feeding habits of sedentary ciliates. Most of sedentary ciliates are filter-feeding predators. They feed mainly on free-living bacteria in water (46). The functional bacteria are mainly distributed in the sludge flocs. Thus, the predation of sedentary ciliates on functional bacteria was relatively less. Alternatively, the free-living ciliates are raptorial predators, and can directly ingest sludge flocs (47), showing more interactions with bacteria community in the activated sludge. When the concentration of chlorine disinfectants increased in the experimental groups, the relative abundance of sedentary ciliates decreased, while the relative abundance of free-living ciliates increased (Fig. 5c and d), resulting in the increase of TSS and bacterial number in the reactor effluents (the bacterial number estimated by HPC method in the effluents of four SBR reactors was 2.7 × 10^3^, 5.8 × 10^3^, 5.4 × 10^3^, and 6.7 × 10^3^ CFU/cm^2^, respectively).

There were 103 protozoa and 37 micro-metazoa with significant interactions (*P* < 0.01, Fig. 7b), including 229 positive interactions and 35 negative interactions, while there were only 42 significant interactions between bacteria and micro-metazoa (*P* < 0.01). This demonstrated that micro-metazoa were relatively inefficient at capturing bacteria-sized particles, and their nutrition could only be obtained by feeding on protozoa (48). Among the significant interactions between protozoa and micro-metazoa, only 41 interactions were found between sedentary ciliates and micro-metazoa. This indicated that micro-metazoa preferred to prey on non-sedentary ciliates.

### Environmental and ecological significance

Based on the full-scale WWTP survey and laboratory-scale SBR reactor experiments, the protozoa community in the activated sludge showed a more sensitivity to the chlorine stress in comparison with bacterial community and micro-metazoa community. Most bacteria live in the zoogloeal, and their secreted EPS can provide a protective barrier against external stress (49). Micro-metazoa are higher-grade and multicellular organisms that possess a higher recalcitrance to external stress. However, due to direct exposure to the water environment, most protozoa (mainly sedentary ciliates) are much more vulnerable to external toxicants (16). Therefore, protozoa, especially sedentary ciliates, can be used as early warning indicators for monitoring the pollutant/toxicant shock in the activated sludge systems.

Besides the disinfection effect of chlorine disinfectants, the microfauna community was also influenced by changes in habitat and bacterial community. For example, the loose structure and excessive EPS of activated sludge caused by chlorination impeded the colonization of sedentary ciliates (Fig. 6). Bacteria in the activated sludge had strong interactions with protozoa, and their changes under chlorine stress directly affected protozoan community and even indirectly affected the micro-metazoa community through the food chain (Fig. 7). Therefore, when evaluating the impact of pollutant/toxicant on the microbial community in the activated sludge systems, research should be conducted through multiple aspects rather than limited to the toxic effects of compounds themselves.

In addition, the environmental and ecological risks related to microfauna in the activated sludge caused by high-dose disinfection during the COVID-19 pandemic could not be ignored. The destruction of the stable structure of microfauna community by chlorination, especially the inhibition of sedentary ciliates, was not conducive to the sedimentation of activated sludge flocs, which would lead to more TSS emissions in the effluent. The suspended solids in the effluents are believed to carry a large amount of emerging pollutants (1). The sedentary ciliates are the main predators of free-living bacteria in the activated sludge systems, and the decline of sedentary ciliates caused by chlorination resulted in the increase of bacterial number in the effluents of which many free-living bacteria have been demonstrated as potential pathogens (50). Moreover, chlorination had led to the release of many protozoa and micro-metazoa from the activated sludge systems (increased amoebas and micro-metazoa were observed in the SBR effluents of experimental groups, Fig. S1).

## SUPPLEMENTAL MATERIAL

Supplemental material aem.01517-24-s0001.docx Text S1 to S4, Tables S1 to S4, and Figure S1.

ASM does not own the copyrights to Supplemental Material that may be linked to, or accessed through, an article. The authors have granted ASM a non-exclusive, world-wide license to publish the Supplemental Material files. Please contact the corresponding author directly for reuse.
