# Supplementary material for: Chlorine disinfectant significantly changed microfauna habitat, community structure, and colonization mode in wastewater treatment plants
Source: Appl Environ Microbiol. 2024 Dec 20;91(1):e01517-24. doi: 10.1128/aem.01517-24 (PMC11784432; doi:10.1128/aem.01517-24)
Supplement: Supplemental material — Text S1 to S4, Tables S1 to S4, and Figure S1. [file aem.01517-24-s0001.docx]

**Supporting Information for**

**Chlorine disinfectant significantly changed microfauna habitat, community structure, and** **colonization mode in wastewater treatment plants**

**Yuening Zhong,^1,2^ Yibo Zhang,^1,2^ Qiyue Meng,^1,2^ Haoyu Zhang,^1,2^ Zhenbing Wu,^1,2^ Chenyuan Dang,^1,2^ Jie Fu^1,2,*^**

^1^Hubei Key Laboratory of Multi-media Pollution Cooperative Control in Yangtze Basin, School of Environmental Science and Engineering, Huazhong University of Science and Technology, Wuhan 430074, China

^2^Green Energy Industry Research Centre (GEIRC), Huazhong University of Science and Technology, Wuhan 430074, China

^*^Address correspondence to Jie Fu, jiefu@hust.edu.cn.

**Text S1.** Sludge treatment method for SEM observation

Part of the sludge sample was taken, and rinsed with 1×PBS (phosphate buffered saline) solution (pH=7.4) for 2-3 times. The time for each rinse was 15 min, and sludge was retained by centrifuging at 5000 rpm for 3 min. After removing the supernatant, sludge sample was fixed in 2.5% glutaraldehyde solution at 4 ℃ for 4 h or overnight. Afterwards, sludge sample was rinsed with PBS buffer for three times (gently blow and mix with gun, 15 min each time, centrifuged at 5000 rpm for 3 min), and then dehydrated with 30%, 50%, 70%, 85%, 95%, 100% (twice) (V/V) ethanol gradient (15 min each time, centrifuged at 5000 rpm for 3 min). The alcohol in the sample was then replaced with isoamyl acetate twice for 20 min each time. The treated sludge sample was then freeze-dried.

**Text S2.** EPS extraction method

The extraction of loosely bound EPS (LB-EPS) and tightly bound EPS (TB-EPS) was carried out by thermal extraction method. In brief, 50 mL sludge mixture was taken at the late aeration stage, centrifuged at 10000 r/min for 10 min, and the supernatant was discarded. Then, 0.05% NaCl heated to 70 ℃ was used to dilute the sludge sample to 20 mL, which was oscillated on a vortex oscillator for 1 min, and centrifuged for 10 min at 10000 r/min. The supernatant was collected and passed through 0.22 μm acetate fiber filtration membrane to obtain LB-EPS. After that, the supernatant in the centrifuge tube was discarded, and residual sludge was diluted to 20 mL with 0.05% sodium chloride solution, bathed in water at 60 ℃ for 30 min, and then centrifuged at 10000 r/min for 10 min. The supernatant was passed through 0.22 μm acetate fiber filter membrane to obtain TB-EPS.

**Text S3.** PCR amplification and sequencing

Full length of 16S rRNA and 18S rRNA genes (V1-V9 regions) were amplified by PCR (95 °C for 5 min, followed by 27 cycles at 95 °C for 30 s, 58 °C for 30 s, and 72 °C for 45 s and a final extension at 72 °C for 10 min) using primers 27F (5′-AGRGTTYGATYMTGGCTCAG-3′) and 1492R (5′-RGYTACCTTGTTACGACTT-3′) for 16S rRNA genes, and EukA (5′-AACCTGGTTGATCCTGCCAGT-3′) and EukB (5′-GATCCTTCTGCAGGTTCACCTAC-3′) for 168 rRNA genes, where each sample was coded by a unique barcode with eight-base. PCR reactions using TransStart Fastpfu DNA Polymerase reaction system were performed in triplicate in 20 μL mixture containing 4 μL of 5 × FastPfu buffer, 2 μL of 2.5 mM dNTPs, 0.8 μL of forward and reverse primers (5 μM), 0.4 μL of FastPfu polymerase, and 10 ng of template DNA. Amplicons were extracted from 2% agarose gels and purified using the AxyPrep DNA Gel Extraction Kit (Axygen Biosciences, USA) according to the manufacturer’s instructions.

Referring to the preliminary quantitative results of electrophoresis, the PCR products were detected and quantified by QuantiFluor™-ST system (Promega Corporation, USA), and then mixed in corresponding proportions according to the sequencing volume requirements of each sample. SMRTbell libraries were prepared from the amplified DNA by blunt-ligation according to the manufacturer’s instructions (Pacific Biosciences, USA). Purified SMRTbell libraries from the Zymo and HMP mock communities were sequenced on dedicated PacBio Sequel II 8M cells using the Sequencing Kit 2.0 chemistry. All amplicon sequencing was performed by Shanghai Biozeron Biotechnology Co. Ltd (Shanghai, China).

**Text S4.** Purification method of isolated *Vorticella* from activated sludge

Step one: A 30 mL of wheat extract was added into the petri dish with exchange every 2 days. The petri dish was generally cultured for 4-6 days. After that, a small amount of *Vorticella* at the bottom of the petri dish would grow and multiply rapidly due to the acquisition of more nutrients, but the sticky substances secreted by *Vorticella* themselves and the nutrients in the wheat extract would adhere to the bottom, which were difficult to be removed by changing the culture medium and rinsing with RO water, causing petri dish contamination. Most microfauna were unable to live in the wheat leach, but some protozoa (e.g., *Tetrahymena*) and micro-metazoa (e.g., rotifers) still survived in the petri dish and competed with the *Vorticella* for nutrients, causing decrease in the growth and reproduction rate of *Vorticella*.

Step two: When a large number of *Vorticella* were attached to the bottom of the petri dish, the sticky substances on the bottom of petri dish was scraped with a cell scraper, and nutrient solution was added to maintain the growth and reproduction of *Vorticella*. After this operation, the sticky substance, *Vorticella* or other microfauna at the bottom were changed to a suspended state. The petri dish was shaken with hands after 1 day of static culture. Then the liquid in the petri dish was poured out, and the bottom of the petri dish was rinsed with RO water 2-3 times. After that, the nutrient solution (wheat extract) was poured into the petri dish, still with exchange every 2 days. Keeping a small amount of suspended viscous substance in the nutrient solution could prevent the petri dish from contamination again due to the metabolism of the *Vorticella*.

Step three: In order to obtain relatively pure *Vorticella*, the petri dish needed to be treated again. The liquid in the petri dish was poured out, and the *Vorticella* cells were rinsed with RO water for 2-3 times (this operation could also use 1×PBS, but it might cause a large number of *Vorticella* death after subsequent operations). A 0.05% pancreatic enzyme solution was added to react for 2-5 min. The atrophy of *Vorticella* cells was observed at all along to prevent the excessive influence. When observation of cell lysis of other micro-metazoa or cell atrophy of *Vorticella* in the petri dish under the microscope, the digestion reaction was stopped, the petri dish was rinsed for 2-3 times, and 10 mL of nutrient solution was added. The surface of the petri dish was observed carefully with the microscope, and residual micro-metazoa were scrapped off with a cell scraper. After *Vorticella* were restored, the culture medium was poured out, the petri dish was washed with RO water for 2-3 times again, and 30 mL of 0.45 μm filter-filtered nutrient solution (wheat extract) was added. After these operations, the bottom of the petri dish showed a relatively clean state. If the petri dish was still existed contamination, repeat steps two and three. However, it should be noted that the time interval for each repeat operation should be more than 3 days. After that, the culture medium was changed every 2 days which could keep the petri dish clean for 2-4 days, or after rinsing the petri dish with RO water for 2-3 times, 30 mL mixture of sterilized inorganic medium (0.24 mM MgSO4, 0.24 mM NaCl) and wheat extract (9:1) was added to keep the purification state for up to 7 days, which could be used for colonization experiments.

**Table S1.** Specific composition of synthetic wastewater

| Composition | Content |
| --- | --- |
| CH_3_COONa | 0.512 g/L |
| NH_4_Cl | 0.19 g/L |
| KH_2_PO_4_ | 0.044 g/L |
| NaHCO_3_ | 1.60 g/L |
| MgSO_4_·7H_2_O | 0.10 g/L |
| CaCl_2_ | 0.10 g/L |
| EDTA | 0.35 mg/L |
| ZnSO_4_·7H_2_O | 0.2 mg/L |
| CuSO_4_·5H_2_O | 0.1 mg/L |
| MnSO_4_·7H_2_O | 0.2 mg/L |
| Co (NO_3_)_2_·6H_2_O | 0.09 mg/L |
| H_3_BO_3_ | 0.1 mg/L |
| Na_2_MoO_4_ | 0.1 mg/L |

**Table S2.** Water quality parameters of chlorinated wastewater

|  | Residual chlorine (mg/L) | COD (mg/L) | NH_4_^+^-N (mg/L) | NO_2_^-^-N (mg/L) | NO_3_^-^-N (mg/L) |
| --- | --- | --- | --- | --- | --- |
| Control | 0 | 360.17 | 49.65 | 0.012 | 3.95 |
| eg1 | 0.08 | 362.43 | 49.18 | 0.012 | 4.05 |
| eg2 | 0.11 | 363.62 | 47.65 | 0.012 | 4.10 |
| eg3 | 0.32 | 337.96 | 47.80 | 0.012 | 3.70 |

Abbreviation: COD, chemical oxygen demand; NH_4_^+^-N, ammonium nitrogen; NO_2_^-^-N, nitrite nitrogen, NO_3_^-^-N, nitrate nitrogen.

**Table S3.** Water quality data of influent and effluent from a full-scale WWTP in Wuhan in February of 2019-2021

|  | | 2019 | 2020 | 2021 |
| --- | --- | --- | --- | --- |
| Water inflow (million m^3^/d) | | 0.31 | 0.32 | 0.35 |
| Residual chlorine in influent (mg/L) | | ND | 0.12 | ND |
| COD (mg/L) | Influent | 212.57 | 198.78 | 232.91 |
|  | Effluent | 22.19 | 23.13 | 26.82 |
|  | Removal | 89.56% | 87.97% | 88.48% |
| NH_4_^+^-N (mg/L) | Influent | 18.91 | 24.19 | 24.58 |
|  | Effluent | 0.23 | 0.47 | 0.46 |
|  | Removal | 98.78% | 98.06% | 98.13% |
| TP (mg/L) | Influent | 4.19 | 2.74 | 4.67 |
|  | Effluent | 0.25 | 0.30 | 0.23 |
|  | Removal | 94.03% | 89.05% | 95.07% |

Abbreviation: COD, chemical oxygen demand; ND, not detected; NH_4_^+^-N, ammonium nitrogen; TP, total phosphorus.

**Table S4.** MLSS in the SBR reactors on day 0 and day 20

|  | Day 0 | Day 20 |
| --- | --- | --- |
| Control | 3500 mg/L | 3000 mg/L |
| eg1 | 3500 mg/L | 2600 mg/L |
| eg2 | 3500 mg/L | 2673 mg/L |
| eg3 | 3500 mg/L | 2333 mg/L |

**
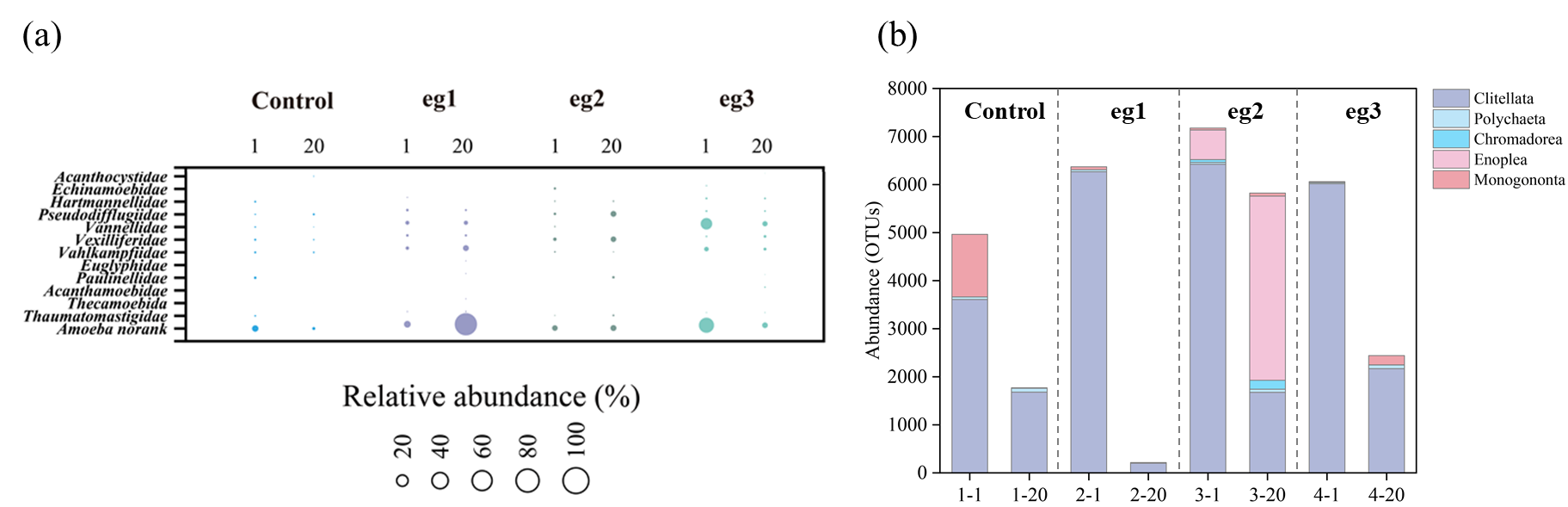
**

**FIG S1** Release of microfauna from the effluents of SBR reactors. (a) Bubble chart of the relative abundance of amoebas at the family level in effluents. (b) Accumulative histogram of abundance of micro-metazoa at class level in effluents.
